# Evaluating Indoor Air Quality in Schools: Is the Indoor Environment a Haven during High Pollution Episodes?

**DOI:** 10.3390/toxics12080564

**Published:** 2024-08-02

**Authors:** Li Sun, Peng Wei, Dane Westerdahl, Jing Xue, Zhi Ning

**Affiliations:** 1 Jiangsu Provincial Key Laboratory of Environmental Engineering, Jiangsu Provincial Academy of Environmental Science, Nanjing 210036, China; sunliapply@126.com; 2 Jiangsu Key Laboratory of Atmospheric Environment Monitoring and Pollution Control, Collaborative Innovation Center of Atmospheric Environment and Equipment Technology, School of Environmental Science and Engineering, Nanjing University of Information Science and Technology, Nanjing 210044, China; 3 Division of Environment and Sustainability, The Hong Kong University of Science and Technology, Hong Kong 999077, China; danewest03@gmail.com; 4 College of Geography and Environment, Shandong Normal University, Jinan 250014, China; pengwei@sdnu.edu.cn; 5 Key Laboratory for Space Bioscience and Biotechnology, School of Life Sciences, Northwestern Polytechnical University, Xi’an 710072, China; xuejing0089@nwpu.edu.cn

**Keywords:** school indoor air, personal exposure, ventilation, AQHI

## Abstract

Pollution data were collected at five schools in Hong Kong using low-cost, sensor-based monitors both indoors and outdoors during two consecutive high pollution episodes. The pollutants monitored included NO_2_, O_3_, PM_2.5_, and PM_10_, which were also used as input to a health risk communication protocol known as Air Quality Health Index (AQHI). CO_2_ was also measured simultaneously. The study aimed to assess the relationship between indoor pollutant concentrations and AQHI levels with those outdoors and to evaluate the efficacy of building operating practices in protecting students from pollution exposure. The results indicate that the regular air quality monitoring stations and outdoor pollutant levels at schools exhibit similar patterns. School AQHI levels indoors were generally lower than those outdoors, with PM_10_ levels showing a larger proportional contribution to the calculated values indoors. NO_2_ levels in one school were in excess of outdoor values. CO_2_ monitored in classrooms commonly exceeded indoor guidelines, suggesting poor ventilation. One school that employed air filtration had lower indoor PM concentrations compared to other schools; however, they were still similar to those outdoors. O_3_ levels indoors were consistently lower than those outdoors. This study underscores the utility of on-site, sensor-based monitoring for assessing the health impacts of indoor and community exposure to urban air pollutants. The findings suggest a need for improved ventilation and more strategic air intake placement to enhance indoor air quality.

## 1. Introduction

Nine million premature deaths in 2015 were reported as being attributable to air pollution by the Lancet Commission on pollution and health [1]. Air pollution is also an important contributor to the non-accidental and cardiovascular mortality of Hong Kong [2]. The Hong Kong Environmental Protection Department (HKEPD) operates 15 general and 3 roadside fixed-site air quality monitoring stations (AQMSs) in Hong Kong for routine air quality monitoring. These stations provide data of high quality that serve to establish the air quality conditions across urban and remote sites. These AQMSs also provide support for the determination of compliance with air quality goals, examine the success of control programs, and to inform the public of possible health risks due to exposure. Most air regulatory programs across the globe summarize and report air quality data in terms of simple concentrations of specific pollutants with a given time-averaging base that can be compared to limits, which are often called ambient air quality standards or guidelines. To better communicate with the public, the HKEPD launched the air quality health index (AQHI) in 2014 to express the short-term health risks of air pollution and to assist the public in taking precautionary measures when ambient pollutant concentrations are high and may cause acute negative health effects. The AQHI is calculated based on the concentrations of nitrogen dioxide (NO_2_), ozone (O_3_), sulfur dioxide (SO_2_), and particulate matter (PM_2.5_ or PM_10_) reported from the AQMSs, and their corresponding calculated health risk indices are based on those values and dose–response algorithms [3]. The AQHI is indexed on a scale of 1 to 10 and 10+ and is reported to the public together with the associated health risks, which are categorized from low (AQHI ≤ 3) and moderate (4 ≤ AQHI ≤ 6) to high (AQHI = 7), very high (8 ≤ AQHI ≤ 10), and serious (AQHI = 10+). According to the HKEPD’s record on AQHI values observed over the 10 years spaning 2014–2023, high pollution episodes (AQHI ≥ 7) commonly occurred in late summer during September and October, and also occurred during the wintertime of January and February [4]. Two high pollution episodes occurred in Hong Kong in succession on 12 September 2017 (Tuesday) and from 16 September (Saturday) to 17 September 2017 (Sunday) during our school-based air quality monitoring campaign from 9 September to 22 September 2017. The reported averaged AQHI by all the AQMSs in Hong Kong had consecutive 8 h, 11 h, and 12 h AQHI above 7 on 12 September, 16 September, and 17 September, respectively. During the events, 10+ was even reported for several hours for most of AQMSs. When AQHI is 7 or above, which is related to high health risks, sensitive groups, such as children and the elderly, are recommended to reduce or minimize outdoor activities [5]. However, whether the indoor environments are safe havens is unconfirmed.

Students are one of the vulnerable populations that are impacted by air pollution; thus, their exposure to harmful air pollution is always a concern. They typically spend one-third to half of their day in school. The personal total daily exposure of school children has been found to be highly correlated to the indoor pollutant concentrations of classrooms [6]. Increased school absenteeism due to respiratory infections and the findings of decreased respiratory functions in children from schools in the most polluted communities have also been associated with elevated air pollution levels [7]. Compared to other indoor environments such as homes, which are often equipped with cooking stoves or other appliances that may be potential indoor pollution sources, school environments have limited localized indoor sources. Our previous study in Hong Kong and studies in other places reported high correlations between school indoor and outdoor air quality [8,9]. A number of studies have been conducted to assess the indoor air quality in schools [10,11,12,13,14,15,16,17,18,19]. Higher levels of PM and NO_2_ levels have been found in some of the classrooms than outdoors in previous studies. Hence, during high pollution episodes, when the outdoor air quality is severe, it remains an open question whether the school microenvironments are safe and healthy places for students. Understanding their nature, especially during such episodes, is crucial for school manager and parents to take proper actions to prevent the excessive exposure of children to air pollution.

Taking advantage of the existing air monitoring campaign that features sensor-based platforms in five schools of Hong Kong during high pollution episodes, air concentration data of NO_2_, O_3_, PM_10_, and PM_2.5_ and environmental data, including carbon dioxide (CO_2_), temperature, and relative humidity (RH), were all collected both indoors and outdoors at the schools. We aimed to comprehensively analyze and understand various aspects of air quality in these environments: (1) characterize the ambient air quality during high pollution episodes, (2) evaluate the relationship between indoor and outdoor air pollutants at schools and its influencing factors, (3) compare the indoor and outdoor AQHI and identify the individual contribution to overall AQHI by pollutant components, and (4) evaluate the nature of ventilation in classrooms and other indoor locations in schools.

## 2. Methods

### 2.1. Site Description

**Figure 1:**
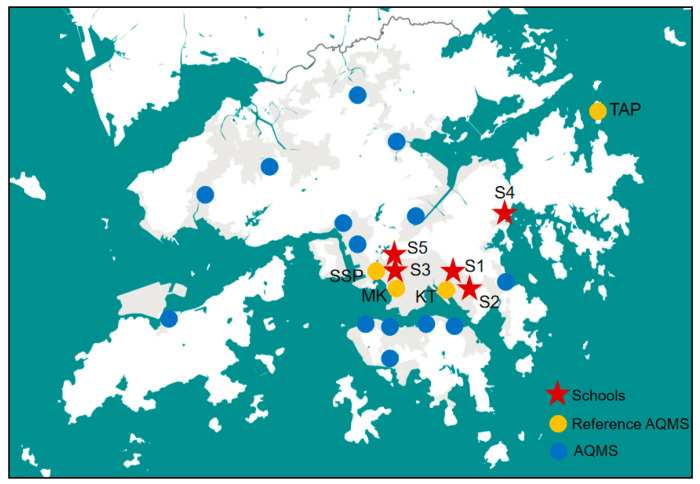
Locations of the investigated schools and AMQSs. The red stars represent the investigated schools, the yellow dots indicate the AQMSs employed for comparison, and the blue dots are the other AQMSs running in Hong Kong.

Five schools (S1, S2, S3, S4, and S5) were under air quality investigation during high pollution episodes occurring in Hong Kong in September of 2017. S1 is built on a hill about 15 m above a secondary road in the Kwun Tong (KT) area of Hong Kong. There is a parking lot in service nearby. S2 is also located on a hill about 20 m above a secondary road in the KT area. It is surrounded by high-rise residential buildings. S3 is situated in the Kowloon area, and it is built next to a railway line. S4 is also located on a hill in a rural area of Hong Kong, and it is away from any heavily trafficked roads. S5 is in a residential area in Kowloon in Hong Kong, and it is in close proximity to secondary roads. The locations of the investigated schools as well as the nearby AQMSs are indicated in Figure 1.

**Figure 2:**
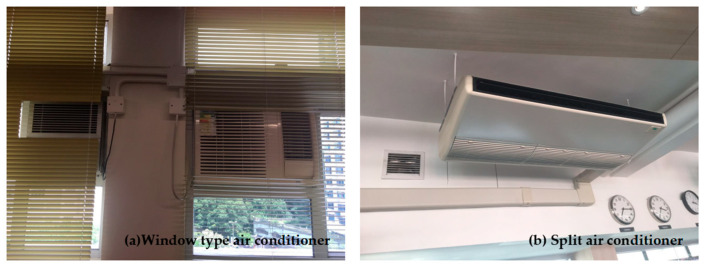
(**a**) Window-type air conditioner and (**b**) split air conditioner.

S1, S3, and S4 utilize window-type air conditioning systems for cooling during summertime, as shown in Figure 2a. In S2, a ductless split-wall-type system is utilized as the air conditioner, as shown in Figure 2b. S5 employs the central air conditioning system, which is equipped with particular filters to remove particles from the fresh air intake before it is introduced into the classroom. Detailed information of each school and sampling sites are summarized in Table 1. It is important to mention that, since the measurement was conducted in the summertime of Hong Kong, windows and doors were commonly closed and air conditioning system was always in operation during school times.

### 2.2. Instrumentation and Measurement Protocol

Two sensor-based monitoring systems, the Mini Air Station (MAS) and the Portable Air Station (PAS) (Sapiens, Hong Kong), were employed for the concurrent measurement of NO_2_, O_3_, CO_2_, PM_2.5_, PM_10_, temperature, and RH. The MAS and the PAS employ the same gas sensor module, which includes an electrochemical NO_2_ sensor and electrochemical O_3_ sensor from Alphasense, Braintree, UK; a PID CO_2_ sensor from Dynament, Mansfield, UK; and a digital temperature and RH sensor from Sensirion, Staefa, Switzerland. The MAS used the model 212 Ambient Particulate Profiler as the PM sensor module, while the PAS employed the Aerocet 831 Handheld Particle Counter because of the space limitation. Both were from Metone, Grants Pass, OR, USA. The MAS is designed in a waterproof case for outdoor measurement. The PAS is fitted into a portable wheeled hard plastic case for both stationary indoor measurement and mobile measurement. Both MAS and PAS can run by either DC power or an inbuilt battery. Outside air was introduced through the gas sensor module by a pump with the flow rate of 1.5 LPM. The original data resolution was set as 10 s. Data were sent to a cloud server in our laboratory via 4G cellular protocols in real time and stored in an internal SD card in the case of the data loss. More information regarding the MAS and the PAS has been described elsewhere [20]. The NO_2_, O_3_, PM_2.5_, and PM_10_ pollutants monitored by the MAS and the PAS were all pre-calibrated before the campaign by co-location with a roadside AQMS. Details of the quality assurance and quality control are described in our previous studies [21,22]. Measurement data from the MAS and the PAS were averaged to a 1 h basis for data interpretation and comparison with conventional AQMS data. Due to the relative low concentrations of SO_2_ pollution in the urban environment of Hong Kong, which almost reached the lower detection limit of SO_2_ sensor, and the negligible contribution of SO_2_ to the AQHI, SO_2_ measurement was not included in the school monitoring campaign.

In each school, two to three indoor sites were chosen for indoor air quality monitoring and at least one classroom was included. For S1, S4, and S5, due to the highly frequent routine usage, the auditorium in schools were also included as indoor sites. For safety reasons, the indoor sampling devices were deployed on the floor and kept as far as possible from the black board where chalk was employed, with the sampling inlet placed approximately 0.6 m from ground to reduce the impact from near-surface turbulence. One outdoor location was selected at each school, typically in a well-ventilated outdoor area, such as a playground, to measure outdoor concentrations simultaneously with indoor concentrations. The sampling period for each school was about 6–7 days including weekdays and weekends, as listed explicitly in Table 1. Due to the limitation on the number of the MAS and the PAS, all the 5 schools were not monitored simultaneously. The air quality monitoring in S1 and S2 encountered the first high pollution episode on 12 September 2017 (Tuesday), while the air quality monitoring in the other three schools encountered the second high pollution episode from 16 September (Saturday) to 17 September 2017 (Sunday).

### 2.3. AQMS Data

The KT community AQMS was located less than 2 km from S1 and S2, and the Sham Shui Po (SSP) community AQMS was also situated 2 km from S3 and S5. These AQMSs provided data that were employed for air quality comparison with school outdoor sampling data. A roadside AQMS, Mong Kok (MK) AQMS, and a background AQMS on Tap Mun (TAP) island were also included for the better interpretation of the high pollution episodes over Hong Kong. The locations of all AQMS sites are shown on Figure 1. The air quality monitoring data of these four reference AQMSs were collected during a 13-day period from 10:00 9 September to 23:59 22 September 2017.

### 2.4. AQHI Calculation

The formulation of Hong Kong AQHI was conducted in 2012 following the original method developed in Canada [3]. It is an expression of health risk and is determined from the sum of the added health risk of the hospital admission attributable to the concentrations of NO_2_, O_3_, SO_2_, and PM_2.5_/PM_10_ on 3 h moving average basis. PM_2.5_ was not included since it was not ubiquitously measured by the AQMSs in Hong Kong during the period when the original AQHI protocols were developed. The AQHI in Hong Kong as calculated by the HKEPD is released hourly to the public. Detailed information regarding the origin and calculations of AQHI can be accessed on the HKEPD website [23] and from a relevant research study [3]. AQHI is routinely communicated based on the results of outdoor regulatory monitoring. In this case, we employed the same methods and made estimates of AQHI from the data collected at various schools. Both the indoor and outdoor AQHIs of schools were calculated following the same formula applied by the HKEPD, except for SO_2_, which was not monitored during the school monitoring campaign.

### 2.5. Ventilation Rate

The ventilation rate (VR) was calculated for the investigated classrooms and indoor spaces using CO_2_ as the tracer gas. The decay, build-up, and steady-state methods are the common methods to indicate ventilation quality through the CO_2_ generated from students indoors. Since Adel Kabirikopaei and Josephine Lau found the steady-state method had less uncertainty than other two methods when considering classroom volume, student CO_2_ emission rate, and other uncertain factors in the calculation [24]. We followed this method to estimate the VR. The calculation equation for the steady-state VR method is shown below:

(1) $$VR=\frac{10^{6}\times G}{C_{steady-state}-C_{outdoor}}$$

where VR is the ventilation rate in the classroom (L/s), G is the CO_2_ generation rate in the classroom (L/s), C_steady-state_ is the indoor steady-state CO_2_ concentration (ppm), and C_outdoor_ is the outdoor CO_2_ concentration (ppm). C_steady-state_ employed the 95th percentile of the 5 min moving average of indoor CO_2_ data in the occupied hours of one day. For each classroom, data of one weekday with typical CO_2_ generation patterns were imported into the equation for VR calculation. The CO_2_ generation rates of students were estimated according to the students’ number, age, and gender. Equal numbers of girls and boys in classrooms were assumed because the exact numbers were not counted. The CO_2_ generation rate applied in this study also referred to the data utilized by Adel Kabirikopaei and Josephine Lau, which were set as 0.244 LPM for S1, S2, and S5 and 0.303 LPM for S3 and S4.

## 3. Results

### 3.1. AQMS Air Quality during the Sampling Period

**Figure 3:**
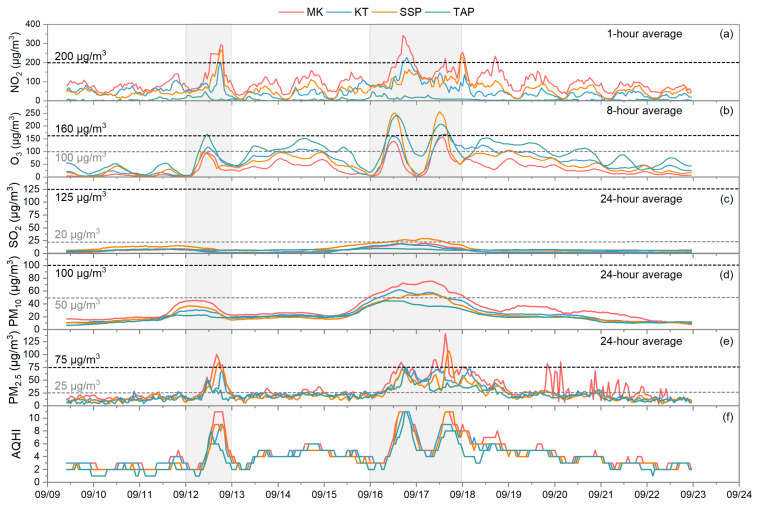
Temporal variation in (**a**) NO_2_, (**b**) O_3_, (**c**) SO_2_, (**d**) PM_10_, (**e**) PM_2.5_ concentrations and (**f**) AQHIs measured by MK roadside AQMS, KT community AQMS, SSP community AQMS, and TAP background AQMS during the period from 10:00 9 September to 23:59 22 September 2017. The shaded parts indicate high pollution episodes. The gray dashed lines represent the 2005 WHO air quality guidelines and the black dashed lines indicate the 2014 HKEPD air quality guidelines.

Temporal variations in the criteria pollutants NO_2_, O_3_, SO_2_, PM_10_, and PM_2.5_ measured at the four reference AQMSs from 10:00 9 September to 23:59 22 September 2017 are shown in Figure 3. Air quality guidelines have been set up by the World Health Organization (WHO) [25] and the HKEPD [26]. The 2005 WHO air quality guidelines and 2014 HKEPD air quality guidelines were adopted in this study since the campaign was conducted in 2017. The WHO air quality guidelines are more stringent, as they define guidelines that have lower concentration values. These serve to help to define safe or acceptable air quality. Both organizations’ guidelines are included in Figure 3 by the dashed lines. Data for the individual pollutants were also averaged in the same temporal resolution following its specific air quality guideline (NO_2_: 1 h; O_3_: 8 h; SO_2_: 24 h; PM_10_: 24 h; and PM_2.5_: 24 h). It is clear in Figure 3 that the concentrations of NO_2_, O_3_, PM_10_, and PM_2.5_ at the roadside site (MK), the community sites (KT and SSP), and the background site (TAP) all experienced dramatic increases during the two high pollution episodes as marked by the gray shades. For NO_2_, the concentrations were generally higher at the roadside site followed by the two general urban sites. The maximum hourly concentration reached about 342 µg/m^3^ during the sampling period. A 1 h concentration limit of 200 µg/m^3^ set by the WHO and the HKEPD was primarily exceeded at the roadside site for 7 h on 12 September, 9 h on 16 September, and 3 h on 17 September. Occasional exceedances also occurred at the community sites. NO_2_ concentrations at the background site varied at a much lower level range (0–50 µg/m^3^) than other sites due to its location on an offshore island without local sources. The ranking of O_3_ concentration levels among the AQMSs displayed the opposite patterns vs. NO_2_. Even though the roadside site had the lowest O_3_ concentration variation ranges among all the four AQMSs, it almost reached the WHO 8 h average standard in the first high pollution episode and surpassed it during the second period. The WHO limits for SO_2_ and PM_10_ were mainly exceeded during the second high pollution episode, while exceedances were observed for PM_2.5_, even on low pollution days.

**Figure 4:**
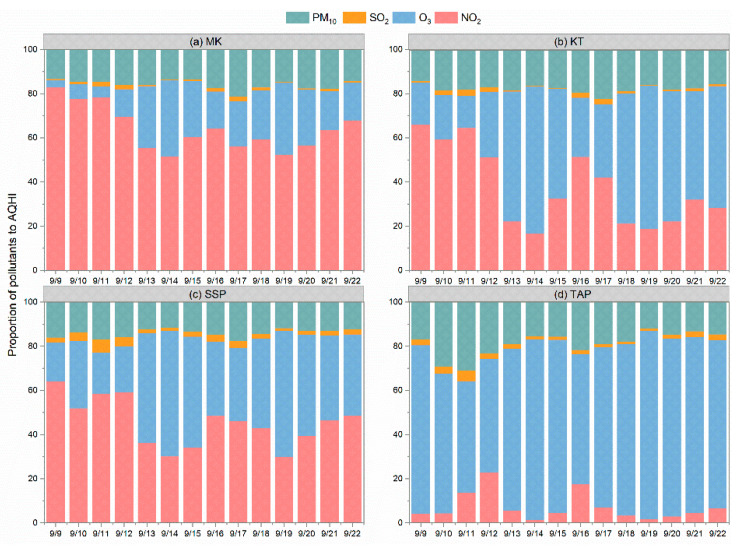
Contribution of NO_2_, O_3_, SO_2_, and PM_10_ to the AQHI on a daily basis calculated from the sampling data of (**a**) MK roadside AQMS, (**b**) KT community AQMS, (**c**) SSP community AQMS, and (**d**) TAP background AQMS.

The hourly AQHIs were then calculated for each of these four AQMSs using the hourly average data and are displayed in Figure 3f. On most days, the AQHI values were mainly lower than 6. However, during high pollution episodes, AQHI markedly increased following the rise in the pollutant concentrations. The data from 35 h to 49 h of the AQHIs were found, based on the AQMSs, to be higher than 7 in the urban environment during those three days. The contributions (%) of the individual pollutants to the added health risk for AQHI formulation were summarized on a daily basis and are listed in Figure 4. It was found that NO_2_ is the major contributor at the roadside, while O_3_ is the major contributor to the background environment. However, O_X_ (NO_2_ + O_3_) account for about 80% of the summed health risk under all the conditions. The remaining 20% was mostly contributed by PM_10_. The proportion of SO_2_ contribution to the AQHI was only 1–3%. Such relative allocation proportions of O_X_, SO_2_, and PM_10_ did not show major changes over the whole sampling period, even during the high pollution episodes.

Overall, AQHI reached high risk, and even serious, designations during the high pollution episodes. The air quality guidelines set by the WHO and the HKEPD were exceeded with the gradually increase in the pollution concentrations, which indicate the potential for more negative health impacts from air pollution on mortality [27]. Moreover, NO_2_ and O_3_ are pollutants that could induce more a acute health impact on the public than SO_2_ and particle pollution in Hong Kong according to their calculated health risk indexes.

### 3.2. Outdoor Air Quality at Schools

**Figure 5:**
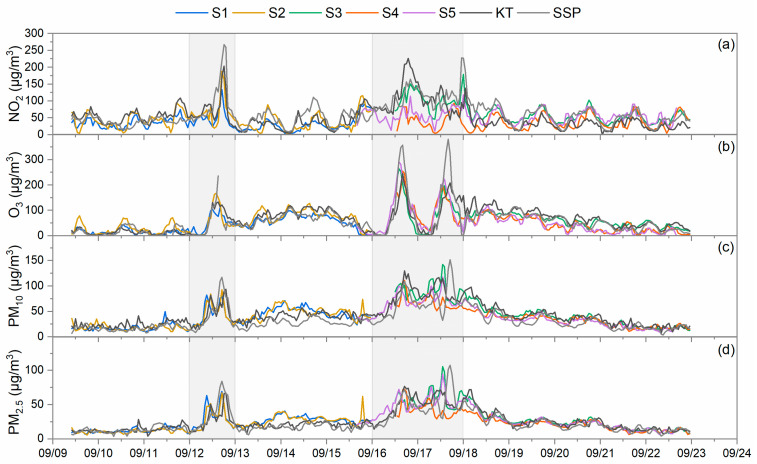
Temporal variation in (**a**) NO_2_, (**b**) O_3_, (**c**) PM_10_, and (**d**) PM_2.5_ concentrations at the outdoor sites of the 5 investigated schools compared to data of KT AQMS and SSP AQMS. The shaded parts indicate high pollution episodes.

The concentrations of NO_2_, O_3_, PM_10_, and PM_2.5_ measured at the outdoor sites of the schools are shown in Figure 5. Two community AQMSs, KT AQMS and SSP AQMS, which are located near to four of the total five investigated schools, were also included in the comparison. The outdoor temporal variation trends for all measured pollutants generally matched well with the AQMSs. This is especially clear for O_3_, PM_10_, and PM_2.5_, for which correlations between the school outdoor environment and AQMS environment were strong with Pearson correlation coefficients (Pearson’s *r*) ranging from 0.74 to 0.95. However, concentration differences were also observed from time to time due to variances in the monitoring location’s unique geography, local meteorological conditions, and local pollution sources. For example, during the second high pollution episode, the average NO_2_ concentrations measured at S4 and S5 were only about 40% and 50% of their corresponding reference AQMS, which degraded the correlations of Pearson’s *r* to 0.31 and 0.45, respectively. In many community exposure studies, data from such AQMSs are commonly used to represent the participants’ outdoor exposure [28]. However, it is clear from this study that the pollution distribution is not homogeneous in the urban environment. The real exposure of individuals or even groups may be overestimated or underestimated especially for source-sensitive pollutants like NO_2_ when using fixed-site AQMSs as a surrogate [29]. The introduction of MAS sensor units in this study for the schools’ outdoor air quality monitoring provides more accurate pollution data to investigate the localized pollutant concentrations observed at the individual schools as well as to evaluate the infiltration of outdoor air into the classrooms.

### 3.3. Indoor Air Quality in Schools

**Figure 6:**
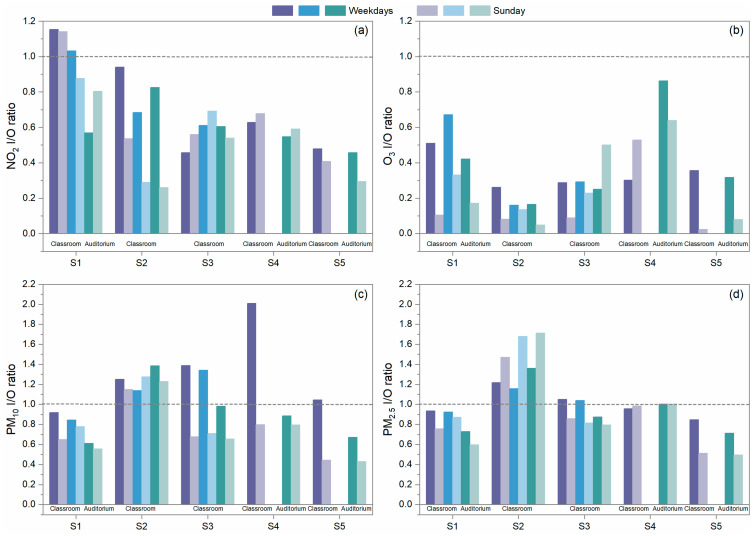
I/O ratio of (**a**) NO_2_, (**b**) O_3_, (**c**) PM_10_, and (**d**) PM_2.5_ calculated from the data collected only during the school time of each investigated school and compared between weekdays and Sundays. S1 has two classrooms (in purple and blue) and one auditorium (in green); S2 and S3 both have three classrooms (in purple, blue, and green); and S4 and S5 both have one classroom (in purple) and one auditorium (in green). Dark purple, blue, and green colors represent the I/O ratio on weekdays; light purple, blue, and green colors represent the I/O ratio on Sundays. The gray dashed lines indicate that the indoor concentration levels are equal to the outdoor ones.

**Figure 7:**
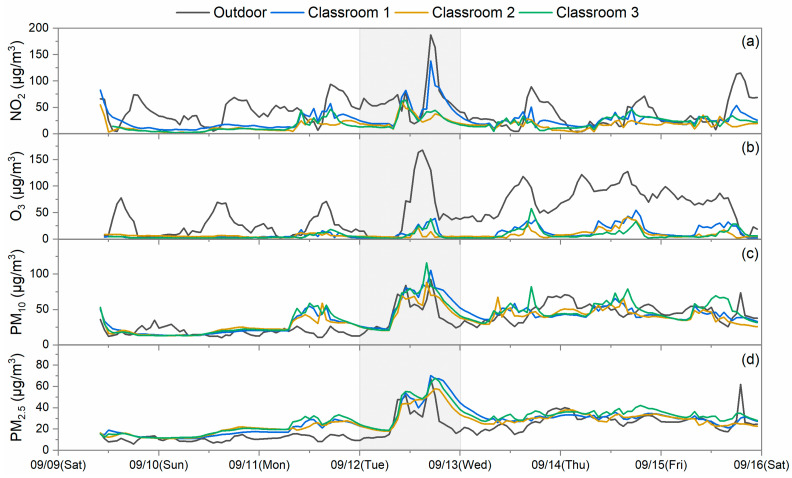
Temporal variation in (**a**) NO_2_, (**b**) O_3_, (**c**) PM_10_, and (**d**) PM_2.5_ measured at the indoor and outdoor sites of S2. The shaded part indicates a high pollution episode.

**Figure 8:**
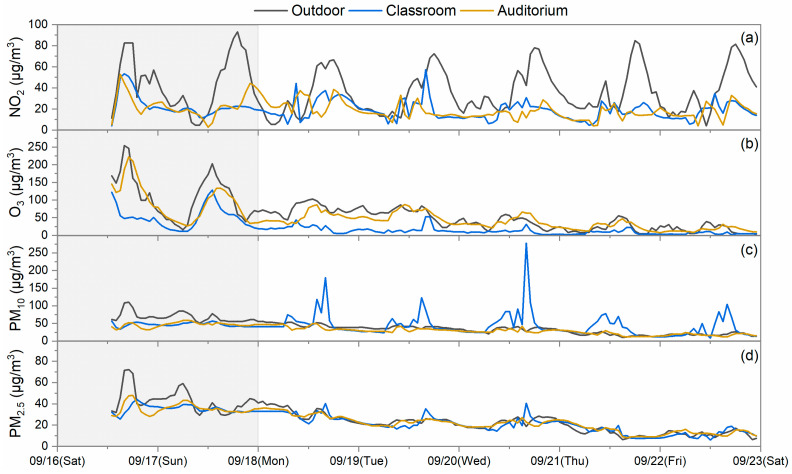
Temporal variation in (**a**) NO_2_, (**b**) O_3_, (**c**) PM_10_, and (**d**) PM_2.5_ measured at the indoor and outdoor sites of S4. The shaded part indicates a high pollution episode.

**Figure 9:**
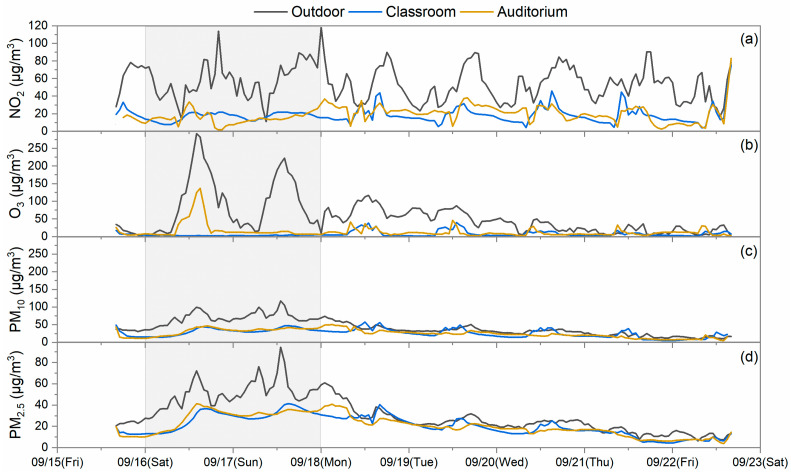
Temporal variation in (**a**) NO_2_, (**b**) O_3_, (**c**) PM_10_, and (**d**) PM_2.5_ measured at the indoor and outdoor sites of S5. The shaded part indicates a high pollution episode.

The outdoor and indoor concentrations of pollutants in the schools were summarized from data collected during the two time periods, which are considered for weekdays and Sunday. Saturday was excluded as students sometimes may have extracurricular activities in the investigated rooms without recorded student numbers and a fixed schedule. Two types of data were collected during the sampling periods based on the classroom operations of weekdays showing operations and occupancy by student vs. Sundays when no students are present. During the summertime in Hong Kong, on weekdays, rooms are occupied by students, and generally, doors and windows are closed for air conditioning operation for the vast majority of the school time, except for the break time. Thus, natural ventilation is limited during summertime. On Sundays, the room is empty, air conditioners are off, and windows are generally kept open. Natural ventilation becomes the dominant contributor to air infiltration. The ventilation rates of rooms depend on the operation of doors, windows, and air conditioning. Indoor vs. outdoor (I/O) concentration ratios were calculated from the grand averages of indoor concentrations and outdoor concentrations collected only during school hours on weekdays and on Sunday too, which are displayed in Figure 6. The temporal variations in the measured pollutants in S2, S4, and S5 are also shown in Figure 7, Figure 8 and Figure 9 as examples to better visualize the correlation of indoor and outdoor pollution. The air quality monitoring performed in S2 was made during the first high pollution episode, while the air quality monitoring in S4 and S5 was in place during the second episode.

It is apparent in Figure 7, Figure 8 and Figure 9 that the indoor NO_2_ and O_3_ of schools were lower in concentration levels than those of the outdoors; however, they somewhat followed the outdoor concentration trends on the sampling days. Relatively low and flat concentration levels of O_3_ were observed from 9 September to 12 September for S2 and on 17 September for S5, which may reflect the impact of reactions occurring between O_3_ and certain chemicals or the limited ventilation in this school during the monitoring. Further research is needed to more completely understand such observations. For S2 and S4, indoor PM pollutants had comparable concentration levels to the outdoor ones, and their trends were also similar. However, for S5, indoor PM pollutants had lower concentration levels compared to the outdoor ones, which was likely due to the particulate matter filters employed in the air conditioning system. The correlation of indoor and outdoor pollutant levels demonstrated moderate to strong correlations between indoor and outdoor pollutants at some indoor sites. For example, Pearson’s *r* for O_3_ of one classroom in S1 was 0.82; that for PM_2.5_ of three classrooms in S2 ranged from 0.72 to 0.77; that for NO_2_ of one classroom in S3 was 0.81; and that for the PM_10_ of the auditorium in S4 was 0.87. These values strongly suggest that outdoor pollution is the driving force in the observed school indoor environments. However, variances in the correlations were also observed among the schools, which mainly depend on the infiltration rates of outdoor pollutants, emissions from indoor sources, and the indoor pollutant decay rates of each school [30].

For the gaseous pollutants NO_2_ and O_3_, I/O ratios were mostly lower than 1 on both weekdays and Sunday during the school time of the sampling periods. NO_2_ I/O ratios on weekdays varied but averaged at 0.69 ± 0.23 (average ± standard deviation). Relatively higher NO_2_ I/O ratios were seen in S1’s classrooms, being larger than 0.8 and even 1. This may be due to the penetration of NO_2_ emitted by the nearby parking lot, which was situated at the other side of the school building and not covered by the outdoor MAS at the air intake of the investigated classrooms. The differences between weekdays and Sunday NO_2_ I/O ratios were generally small. This indicates that NO_2_ can penetrate indoors almost equally through mechanical ventilation and natural ventilation from outdoor sources. The largest difference was identified in S2. This may be because the doors and windows of S2 were fully closed during the weekends in the absence of mechanical ventilation. Thus, the penetration pathway from outdoor to indoor was strictly restricted, which led to low concentration levels of NO_2_ indoors. Since O_3_ is a reactive pollutant, which can readily react with building materials and other products (e.g., cleaning chemicals) [9], the I/O ratios of O_3_ on weekdays were typically lower than those of NO_2_, varying around 0.37 ± 0.20. However, relatively higher O_3_ I/O ratios (>0.6) were detected in S4’s auditorium on both weekdays and the weekend. This may be because the ventilation at that location was in a mixed ventilation mode dominated by natural ventilation, which indicates the possibility for O_3_ to have a higher I/O ratio in the pure natural ventilation condition.

The I/O ratios of PM_10_ and PM_2.5_ differed from those of the gases. The PM_10_ I/O ratios were found to be higher on weekdays when students were present, and they were mostly close to or even higher than 1, which averaged at 1.11 ± 0.37. This phenomenon has also been found in other studies performed in schools [18,31]. Particles from outdoors can penetrate into the indoors through ventilation and the building envelope with minor losses during the process. Moreover, particles indoors with diameters larger than 5 µm can be easily re-suspended by the activities in the rooms [32]. Thus, occasional sharp increases in PM_10_ were observed in classrooms of S3 and S4 (the S4 data are displayed in Figure 8), and ultimately, led to higher I/O ratios. PM_2.5_ also had comparable levels of I/O ratios with PM_10_ averaging at 0.99 ± 0.19, which was due to both indoor and outdoor sources [33]. A similar observation was also presented in other school study [34]. Reductions in PM_2.5_ and PM_10_ I/O ratio levels were also observed on Sundays compared to weekdays because of the absence of some indoor sources. After penetration from the outdoors and generation from the indoors, PM_2.5_ could stay in a stable and suspended condition. Sometimes, it even took a longer time for them to follow the decay trend of outdoor PM_2.5_ after reaching the concentration peak as observed in S2 and demonstrated in Figure 7. This also raises our concern that more exceedances of the air quality guidelines may occur indoors than outdoors. S5 installed a high efficiency particle filter in their ventilation duct to pre-condition the fresh air before it enters the air conditioning system. It did lead to somewhat lower I/O ratios compared to those of other schools. However, the classroom still had an I/O ratio larger than the unity on weekdays. It seems like the current precaution measure of S5 is not sufficient to produce promising efficacy.

### 3.4. School AQHIs

**Figure 10:**
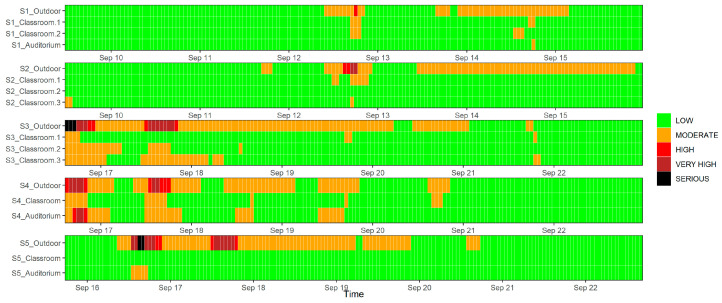
Temporal variation in the AQHI calculated from the indoor and outdoor pollutant concentrations of 5 schools during individual air quality monitoring periods.

Indoor and outdoor AQHIs of schools were all calculated hourly using the 3 h moving average data of NO_2_, O_3_, and PM_10_ concentrations, which are visualized in Figure 10 by the AQHI’s corresponding colors indicated by the HKEPD. Green represents an AQHI of 1–3; orange equals to an AQHI of 4–6; red indicates an AQHI of 7; brown is an AQHI in the range of 8–10; while black means the AQHI is even higher than 10. It is apparent that the high pollution episodes also influenced the school outdoor environments, though specific outdoor AQHI values at S1, S2, S4, and S5 may be one or two indexes lower than those released by the reference AQMSs. This is mainly due to the lower concentration levels of NO_2_ and O_3_ at some school sites. In most investigated indoor environments of schools, the indoor AQHI was in low and moderate levels. This may be driven by the partial reduction in NO_2_ concentrations and the greater removal of O_3_ pollution during the infiltration process from outdoors to indoors in summertime when air conditioning is operated. Only one auditorium of S4 experienced an indoor AQHI at high and very high levels, which was likely driven by the high penetration of O_3_ pollution under largely open ventilation conditions. It is important to remember that this study was conducted when air conditioning was in use. It is possible that different conditions might be found for indoor environments during cooler seasons when natural ventilation is more commonly used. Doors and windows are often left open for better air circulation during class time during these periods.

**Figure 11:**
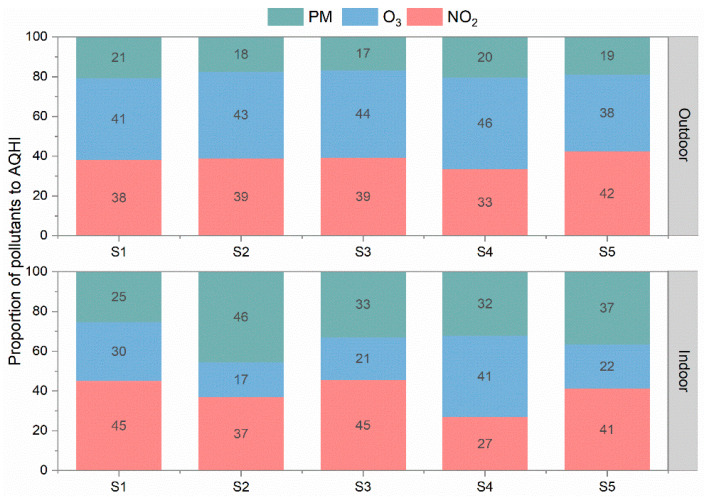
Contributions of NO_2_, O_3_, and PM_10_ to the indoor and outdoor AQHIs of 5 schools.

The contributions of NO_2_, O_3_, and PM_10_ pollutants to the outdoor AQHI and indoor AQHI were summarized for each school through the entire monitoring period and are displayed in Figure 11. For the outdoor AQHI calculated from the school monitoring, the proportions of NO_2_, O_3_, and PM_10_ contributions matched with those of the urban AQMS sites, where O_X_ accounted for 80% while PM_10_ covered the remaining 20%. However, in the indoor environments we studied during the summertime, the contribution from PM_10_ increased to more than 30% in S3, S4, and S5 and the maximum was about 46% in S2. Higher indoor particle concentration levels than outdoor levels were observed under both mechanical ventilation and natural ventilation, even in the absence of student activities. This implies that more care should be paid to indoor particle pollutions. Actions are needed to reduce indoor PM, which might include improved room cleaning and perhaps added filtration.

### 3.5. School Ventilation Rates

**Figure 12:**
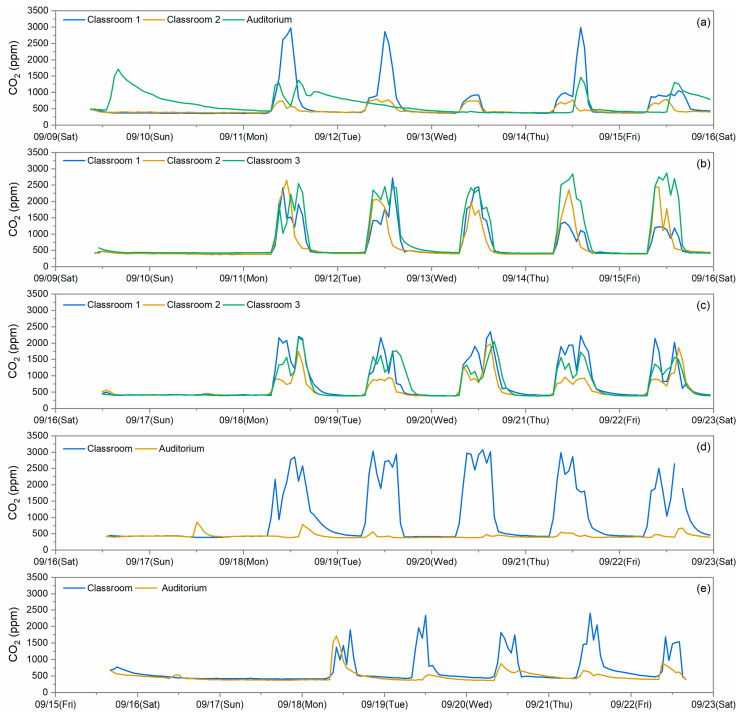
Temporal variation in CO_2_ concentrations measured at the indoor sites of (**a**) S1, (**b**) S2, (**c**) S3, (**d**) S4, and (**e**) S5.

During summertime in Hong Kong, doors and windows are mostly closed. Air conditioners are used during the periods when the rooms are occupied with students. In this study, CO_2_ was used as a tracer gas to indicate the ventilation conditions of indoor environment. CO_2_ concentrations were measured with other pollutants both indoors and outdoors, and time series observations are shown in Figure 12. The outdoor CO_2_ concentration levels were fairly constant and below 500 ppm during the sampling period, while indoor CO_2_ concentrations are shown to have marked peak periods that occur when spaces are occupied by the students. Its concentration increased sharply after the students entered the room in the morning; then, it stayed at a relative high level (reaching at steady state) during the class time. The CO_2_ levels dropped back to the ambient levels when students left at the end of the school day. The CO_2_ concentrations were seen to build up to 2000–3000 ppm levels in the majority of the measured indoor environments, which is much higher than the standard 62.1 ventilation requirements of 1000 ppm set for classrooms by the American Society of Heating, Refrigeration and Air Conditioning Engineers (ASHRAE) [35]. The VRs per person were calculated only for classrooms using the indoor steady-state CO_2_ concentrations and are listed in Table 2. The VR was not calculated for the investigated auditorium because of the fluctuation in student numbers. Most of the classrooms had VRs in the range of 1–4 L/s/person, which are lower than the minimum VR required by the ASHRAE standard 62.1 of 6.7 L/s/person for students older than 9 years old and 7.4 L/s/person for students aged 5–8 years. Only two classrooms showed VRs higher than the requirement during the sampling period. This means that, even though the mechanical ventilation was in use when the rooms were occupied, it did not always provide sufficient ventilation to the students. Inadequate ventilation is a very common problem for classrooms [36,37]. Studies have shown associations between increased indoor CO_2_ levels and certain sick building syndrome (SBS), like headache, dizziness, and difficulty in concentrating [38], and it also has a certain impact on students’ productivity [39]. Higher VRs have been shown to have the potential to reduce students’ illness and absenteeism [40]. Hence, these findings strongly support the need to assess and perhaps improve ventilation in the classrooms of Hong Kong.

## 4. Discussion

This paper reports on the observations from a sensor-based air quality measurement campaign conducted at five schools in Hong Kong for measuring NO_2_, O_3_, CO_2_, PM_10_, and PM_2.5_ in both outdoor and indoor environments. The campaign coincided with two significant pollution episodes, which provided a unique opportunity to examine the dynamics between indoor and outdoor air quality and assess the effectiveness of conventional advice to remain indoors during such episodes in reducing health risks from outdoor pollutants. Furthermore, this offered an opportunity to observe whether the conventional guidance to stay indoors is adequate to reduce health risks from exposure to pollutants of outdoor origin and provide full safeguarding as intended during high pollution episodes. Indoor air quality in high pollution episodes is not well studied in Hong Kong. Thus, this study demonstrates the potential applicability of on-site monitoring that employs sensor-based protocols, when used in conjunction with AQMSs, in determining the possible health risks to indoor occupants and community associated with exposure to common urban air pollutants. It also provides a strong basis for concern regarding practical issues linked to closed indoor environments, such as schools.

During the air quality monitoring in schools, indoor school environments had reduced NO_2_ and O_3_ concentrations compared to outdoor levels, which may be attributed to the gas loss due to the chemical reaction with and deposition on the object surface during the infiltration [41]. Since O_3_ is a more active pollutant, it can easily react with reductive chemicals, which ultimately induces a higher removal of O_3_ than NO_2_ during the penetration process. However, elevated PM_10_ concentration levels were captured indoors due to the re-entrainment of larger particles by the movement of students or chalks used by teachers. PM_2.5_ indoor concentrations were also found to be similar to those outdoors. Overall, the indoor AQHI levels mainly ranged within acceptable low and moderate ranges, which are an attribute to the dramatic decrease in gas pollutant levels indoors from the usage of mechanical ventilation during the summertime. However, one exception occurred in one of the investigated auditoriums during the weekend. The high infiltration of O_3_ under natural ventilation induced high and very high levels of indoor AQHI. It was found that NO_2_ does not have a favored ventilation mode; however, O_3_ prefers the natural ventilation mode. This raised our concerns that there may be a higher chance to have an endangering indoor environment in other seasons, like spring and fall, when opening windows and doors is the routine operation. Insufficient ventilation was also observed in classrooms from the collected CO_2_ data. Thus, refinements may be needed to both reduce pollutants from outside air while also reducing the CO_2_ generated by the students. However, while increasing ventilation rates would reduce CO_2_ levels, it could bring in more polluted outdoor air and could require system modifications and increased operation costs. The site of air intake should also be carefully selected to avoid community sources of pollution, such as proximity to busy roadways. Contributions of pollutants to the AQHI were also analyzed. NO_2_ and O_3_ are the major contributors, accounting for 80% of the AQHI in ambient environment, while the proportion of PM was noted to be increased in the indoor environment. Even though PM contributed a smaller fraction of the AQHI, it is clear that more attention should be paid to particulate pollution indoors in consideration of its close to or even higher than unity I/O ratios.

It should be noted that this study was performed during a period when air conditioning was in use and classrooms were largely kept closed. During other periods in Hong Kong, when the outdoor temperatures are more moderate, windows and doors are often left open and air conditioners are turned off. Hong Kong has major pollutant episodes during these cooler periods where outdoor pollutants are high; especially, PM_2.5_ levels may be at theit highest during the winter months due to the transport of polluted air from other regions of Asia. It is likely that ventilation rates differ from what was observed in this study and that indoor pollutant levels might also be quite different. The resulting AQHI estimations for cooler season classroom-related exposure are also likely to be different from what was observed in this study. The question ‘Is the indoor environment a haven during high pollution episodes?’ remains open. For reactive air pollutants, like O_3_, it does seem to be true. However, for other pollutants, we provide no clear answer. Building ventilation practices were found to be important, and these practices and pollutant conditions differ by season. Thus, further studies of indoor air and its relationship to community and outdoor air would be useful. The use of purpose-built monitoring systems of the type used in this study are particularly suited for such studies due to their relatively low cost and ease of placement and autonomous operations. Future studies of ventilation conditions and how they relate to observed exposures could also clarify how to act to better control indoor exposures.

The implications of these findings are significant for public health policy and building management. They suggest the necessity for refined strategies that balance the reduction in indoor CO_2_ and infiltration of outdoor pollutants. While increasing ventilation can mitigate CO_2_ buildup, it may also introduce more outdoor pollutants unless air intakes are strategically placed away from local pollution sources. Moreover, the economic implications of modifying ventilation systems to handle increased loads must be considered.

In conclusion, this study not only sheds light on the complex interplay between indoor and outdoor air quality, but also establishes a foundation for future research. Further studies should explore the seasonal variability of indoor air quality and its health implications, employing cost-effective and easily deployable sensor-based monitoring systems. These investigations could lead to a more nuanced understanding and management of indoor air pollution across different environmental conditions and building usage patterns.

## Figures and Tables

**Table 1:** Description of the sampling sites at investigated schools.

School ID	School Type	Site Type	Floor	Floor Area */m^2^	Student Number	Ventilation Mode	Potential Outdoor Source	School Time	Sampling Period
S1	Primary School	Outdoor	2/F	NA	NA	NA	Nearby parking lot	8:00–15:00	10:00 9 September–23:59 15 September 2017
		Classroom1	2/F	60	31	Window-type air conditioner			
		Classroom2	5/F	60	30	Window-type air conditioner			
		Auditorium	6/F	190 (7 m)	NA	Air conditioner			
S2	Primary School	Outdoor	G/F	NA	NA	NA	NA	8:00–15:00	10:00 9 September–23:59 15 September 2017
		Classroom1	2/F	70	32	Split air conditioner			
		Classroom2	2/F	70	25	Split air conditioner			
		Classroom3	6/F	70	26	Split air conditioner			
S3	Secondary School	Outdoor	G/F	NA	NA	NA	Nearby railway	8:00–15:00	12:00 16 September–23:59 22 September 2017
		Classroom1	2/F	55	24–30	Window-type air conditioner			
		Classroom2	2/F	55	24–30	Window-type air conditioner			
		Classroom3	5/F	55	24–30	Window-type air conditioner			
S4	Secondary School	Outdoor	G/F	NA	NA	NA	NA	7:30–17:00	12:00 16 September–23:59 22 September 2017
		Classroom	G/F	55	31	Split air conditioner			
		Auditorium	2/F	352 (6.5 m)	NA	Split air conditioner			
S5	Primary school	Outdoor	2/F	NA	NA	NA	Adjacent roads	9:00–15:00	16:00 15 September–16:59 22 September 2017
		Classroom	2/F	50	15–25	Split air conditioner with particle filter			
		Auditorium	5/F	450 (7.5 m)	NA	Split air conditioner with particle filter			

NA: Not applicable. * Heights of all the classrooms are about 3 m; Heights of the auditoriums are listed in brackets.

**Table 2:** Ventilation rates of the indoor sampling sites at the investigated schools.

School ID	S1		S2			S3			S4	S5
Classroom No.	Classroom 1	Classroom 2	Classroom 1	Classroom 2	Classroom 3	Classroom 1	Classroom 2	Classroom 3	Classroom	Classroom
VR (L/s/person)	1.55	11.16	1.76	1.67	2.12	2.42	9.30	3.86	2.54	1.16

## Data Availability

The data presented in this study are available upon request from the corresponding author.
